# Prevalence of Cardiovascular-Kidney-Metabolic Syndrome Stages by Social Determinants of Health

**DOI:** 10.1001/jamanetworkopen.2024.45309

**Published:** 2024-11-18

**Authors:** Ruixin Zhu, Ran Wang, Jingjing He, Langrun Wang, Huiyu Chen, Xiaokang Niu, You Sun, Yiran Guan, Yifan Gong, Liwei Zhang, Peng An, Keji Li, Fazheng Ren, Weili Xu, Jie Guo

**Affiliations:** 1 Key Laboratory of Precision Nutrition and Food Quality, Department of Nutrition and Health, China Agricultural University, Beijing, China; 2 College of Food Science and Engineering, Tianjin University of Science and Technology, Tianjin, China; 3 Department of Nutrition and Food Hygiene, School of Public Health, Peking University, Beijing, China; 4 Department of Neurobiology, Care Sciences and Society, Karolinska Institutet, Solna, Sweden

## Abstract

**Importance:**

Cardiovascular-kidney-metabolic (CKM) syndrome—a novel, multistage, multisystem disorder as defined by the American Heart Association—is highly prevalent in the US. However, the prevalence of CKM stages by social determinants of health (SDOH) remains unclear.

**Objective:**

To investigate whether the prevalence of CKM stages varies by SDOH in US adults.

**Design, Setting, and Participants:**

This cross-sectional study used data from the National Health and Nutrition Examination Survey (1999-2018) and included a nationally representative sample of adults aged 30 to 79 years through complex, multistage probability sampling. Data were analyzed from April 1 to June 15, 2024.

**Exposures:**

The exposures included 5 CKM stages (ie, stages 0-4) reflecting progressive pathophysiology, with advanced (stages 3 or 4) and nonadvanced (stages 0, 1, or 2) disease. CKM stages were defined based on risk factors for metabolic syndrome, cardiovascular disease, and chronic kidney disease.

**Main Outcome and Measures:**

The main outcome was the age-standardized prevalence of CKM stages and advanced CKM stages across SDOH, including education, marital status, family income, food security, health insurance, employment, home ownership, and health care access.

**Results:**

Among 29 722 participants (weighted mean [SE] age, 50.8 [0.1] years; weighted 50.7% male), the age-standardized prevalence of CKM stages 0 to 4 was 13.6% (95% CI, 13.0%-14.3%), 29.9% (95% CI, 29.1%-30.7%), 43.7% (95% CI, 42.9%-44.5%), 4.7% (95% CI, 4.4%-5.0%), and 8.1% (95% CI, 7.6%-8.5%), respectively. Significant differences were observed in the prevalence of CKM stages across all unfavorable SDOH of interest compared with their favorable counterparts, with unemployment (18.8% [95% CI, 17.7%-20.1%] vs 11.4% [95% CI, 11.0%-11.9%]), low family income (16.1% [95% CI, 15.4%-16.8%] vs 10.1% [95% CI, 9.5%-10.7%]), and food insecurity (18.3% [95% CI, 17.1%-19.6%] vs 11.7% [95% CI, 11.2%-12.2%]) associated with an increased likelihood of advanced CKM stages. Participants with 2 or more unfavorable SDOH were more likely to have advanced CKM stages (age-standardized prevalence, 15.8% [95% CI, 15.2%-16.5%] vs 10.5% [95% CI, 9.9%-11.1%] with <2 unfavorable SDOH). Living in a rented home (15.9% [95% CI, 14.7%-17.0%] vs 9.3% [95% CI, 8.7%-9.9%] owning the home) or not living with a partner (13.2% [95% CI, 12.3%-14.3%] vs 9.2% [95% CI, 8.5%-9.8%] living with a partner) increased the likelihood of advanced CKM stages in female but not male participants.

**Conclusions and Relevance:**

In this cross-sectional study, disparities in the prevalence of CKM stages by SDOH, particularly family income, food security, and employment, with notable sex differences, were observed in US adults. These findings highlight the need to address inequities in CKM syndrome through targeted interventions.

## Key Points

**Question:**

Does the prevalence of cardiovascular-kidney-metabolic (CKM) syndrome stages vary by social determinants of health (SDOH) in US adults?

**Findings:**

In this cross-sectional study of 29 722 US adults, unemployment, low family income, food insecurity, and having 2 or more unfavorable SDOH were associated with an increased likelihood of advanced stages of CKM. Living in a rented home or not living with a partner were associated with an increased likelihood of advanced CKM stages among females but not males.

**Meaning:**

These findings suggest a disproportionate burden of CKM syndrome by SDOH and sex and underscore the urgent need to address these inequities.

## Teaser

This cross-sectional study investigates whether the prevalence of cardiovascular-kidney-metabolic syndrome varies by social determinants of health among US adults.

## Introduction

Cardiovascular-kidney-metabolic (CKM) syndrome, recently defined by the American Heart Association (AHA), describes the interplay among obesity, diabetes, chronic kidney disease (CKD), and cardiovascular disease (CVD).^1^ To capture the progressive nature of CKM syndrome, the AHA introduced a staging framework ranging from stages 0 to 4.^1^ Recent studies indicated that CKM syndrome is highly prevalent among US adults, with approximately 90% meeting the criteria for CKM stage 1 or higher between 2011 and 2020.^2^ Notably, 15% of US adults are classified as having advanced CKM stages (stages 3 or 4), with significant differences in prevalence based on sex and age.^2^ Specifically, men are more likely to have advanced CKM stages than women, while advanced CKM stages are more common in older adults (aged ≥65 years) than younger adults (aged 20-44 years).^2^

Beyond age and sex, social determinants of health (SDOH), which are indicators of health equity, are associated with health outcomes. SDOH refer to nonmedical risk factors such as income, education, employment, housing, food security, and access to affordable health services.^3^ The US Healthy People 2030 initiative aims to achieve health equity and improve well-being through addressing SDOH.^4^ Previous studies have shown that SDOH significantly influence the development, diagnosis, and outcomes of the major components of CKM syndrome, such as diabetes, CKD, and CVD.^1,3,5,6,7^ Adverse SDOH have downstream consequences for events and mortality from diabetes, CKD, and CVD through behavioral pathways, such as physical inactivity and unhealthy diets.^1,3,6,8^ Given the interconnected nature of obesity, diabetes, CKD, and CVD within CKM syndrome, adverse SDOH may contribute to the progression of CKM syndrome.

Identifying which SDOH require targeted attention might inform more effective screening and prevention strategies. However, the prevalence of CKM stages across various SDOH remains unclear. The aim of this study was to examine the prevalence of CKM stages 0 to 4 or advanced CKM stages (stages 3 or 4) by SDOH in a large, nationally representative sample of US adults. Additionally, we explored sex differences in the prevalence of advanced CKM stages by SDOH.

## Methods

### Study Population

This cross-sectional study used data from the National Health and Nutrition Examination Survey (NHANES), a serial, cross-sectional, national survey with a complex, stratified, multistage probability design. The NHANES protocol was approved by the National Center for Health Statistics Research Ethics Review Board. All participants or their guardian provided written informed consent prior to data collection. This study was exempt from review because it involved secondary data analysis of deidentified data, which poses minimal risk to participants’ privacy. This study followed the Strengthening the Reporting of Observational Studies in Epidemiology (STROBE) reporting guideline for cross-sectional studies.

The NHANES has been collecting nutritional and health information from a nationally representative sample of noninstitutionalized US adults and children every 2 years since 1999-2000 using a multistage, stratified sampling design.^9^ Response rates for NHANES participants have decreased over time from 76% in 1999-2000 to 49% in 2017-2018.

This study used data from 10 NHANES cycles (from 1999-2000 to 2017-2018). We excluded participants who were pregnant or lactating as well as those whose 10-year CVD risk could not be estimated using the AHA Predicting Risk of CVD Events (PREVENT) equations, which were used for the definition of CKM stages (ie, participants aged <30 years or >79 years or those with missing or extreme values of CVD risk factors) (eFigure 1 in Supplement 1). The final analytic sample comprised participants aged 30 to 79 years.

### CKM Syndrome Stages

CKM syndrome stages (ie, stages 0-4) were defined based on the criteria from Aggarwal et al,^2^ with modifications for NHANES data. Briefly, participants were categorized by CKM stage as follows. Stage 0 included participants with a body mass index (BMI; calculated as weight in kilograms divided by height in meters squared) of 18.5 to 24.9 and waist circumference of less than 88 cm for women or less than 102 cm for men who did not meet criteria for the other stages. Stage 1 included participants with a BMI of 25 or higher, a waist circumference of 88 cm or greater for females or 102 cm or greater for males, or prediabetes (fasting blood glucose of 100-124 mg/dL, glycosylated hemoglobin A_1c_ of 5.7%-6.4%, or taking diabetes medications [to convert glucose to mmol/L, multiply by 0.0555; to convert hemoglobin A_1c_ to proportion of total hemoglobin, multiply by 0.01]). Stage 2 included participants with metabolic risk factors (elevated fasting serum triglyceride levels ≥135 mg/dL [to convert to mmol/L, multiply by 0.0113], hypertension, diabetes, or metabolic syndrome) or moderate- to high-risk CKD according to Kidney Disease: Improving Global Outcomes criteria.^10^ Stage 3 included participants with very-high-risk CKD by Kidney Disease: Improving Global Outcomes criteria or a high predicted 10-year CVD risk. Stage 4 included participants with self-reported CVD (coronary heart disease, angina, heart attack, heart failure, or stroke). The 10-year CVD risk was estimated using the AHA PREVENT equations suitable for individuals aged 30 to 79 years.^11^ Participants were also classified into advanced (stages 3 or 4) or nonadvanced (stages 0, 1, or 2) CKM stages. High CVD risk was defined as a 10-year risk of at least 20% as estimated using the PREVENT equations. The CKD stages were classified based on estimated glomerular filtration rate (calculated using the race-free Chronic Kidney Disease Epidemiology Collaboration 2021 creatinine equation^12^) and urinary albumin to creatinine ratio.

### Social Determinants of Health

The SDOH of interest were dichotomized into favorable vs unfavorable conditions according to the conventional cut points: employment status (employed, student, or retired vs unemployed), family income to poverty ratio (≥300% vs <300%), food security (full security vs marginal, low, or very low security), education (high school graduate or higher vs less than high school), health care access (at least 1 regular health care facility vs none or emergency department), health insurance status (private vs government or no insurance), home ownership (own home vs rent home or other arrangement), and marital status (married or living with a partner vs not).^13^ Cumulative unfavorable SDOH were calculated based on the number of unfavorable SDOH domains (range, 0-8) and were dichotomized as 2 or more vs fewer than 2 (2 being the median value of the number of unfavorable SDOH domains).^13^

### Potential Confounders

Potential confounders, including age (continuous variable), sex (female or male), race and ethnicity (Mexican American; non-Hispanic Black, non-Hispanic White, and other [other Hispanic or other race, including multiracial and any race other than Black or White]), smoking status (current, former, or never smoker), alcohol consumption (nondrinker, <12 alcohol drinks per year; moderate drinker, ≥12 drinks per year but <1 drink per day for females or <2 drinks per day for males; heavy drinker, ≥1 drink per day for females or ≥2 drinks per day for males), and physical activity (active, ≥150 minutes per week of moderate-intensity activity or ≥75 minutes per week of vigorous-intensity activity; inactive, less than these thresholds) were collected during the household interview and self-reported by participants in each NHANES cycle using standard questionnaires. Race and ethnicity were self-identified by participants. For analytic purposes, other Hispanic and other races were combined due to their limited sample size. Dietary factors were excluded from the analysis due to prevalent misreporting of energy and nutrient intake in NHANES data.^14,15^

### Statistical Analysis

Statistical analyses were performed using Stata, version 18.0 (StataCorp LLC) and SPSS, version 28.0 (IBM Corporation) from April 1 to June 15, 2024. Data were adjusted for the complex sampling survey design of NHANES, with strata, primary sampling units, and probability weights incorporated into statistical models using survey analysis procedures.^16^ Differences in baseline characteristics across the 5 CKM stages and between advanced (stages 3 or 4) and nonadvanced (stages 0, 1, or 2) CKM stages were assessed using survey-weighted linear regression for continuous variables and survey-weighted Pearson χ^2^ tests for categorical variables.

For the main analyses, data from NHANES 1999-2018 and 20-year sampling weights of 10 survey cycles were used. Age-standardized CKM prevalence rates were calculated by the direct standardization method with 3 age categories, including 30 to 44 years, 45 to 64 years, and 65 to 79 years, as in:
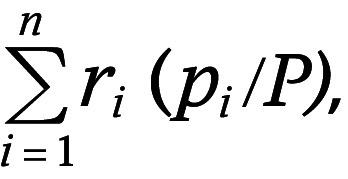
where *r_i_* is the rate in age group *i* in the population of interest, *p_i_* is the standard population in age group *i*, *P* is ∑*^n^_i_*_ = 1_, and *n* is the total number of age groups over the age range of the age-adjusted rate.

The 2010 US census population was selected as a standard population.^16,17^ The 95% CIs were estimated using Taylor series linearization.

Differences in the prevalence of CKM stages across age groups, sexes, races and ethnicities, and SDOH were determined using survey-weighted and age-standardized Pearson χ^2^ test. Survey-weighted multinomial logistic regression models, adjusted for baseline age, sex, race and ethnicity, alcohol consumption, smoking status, and physical activity, were used to estimate relative risk ratios (RRRs) and 95% CIs for the associations between SDOH and the prevalence of CKM stages. The multinomial logistic regression model was chosen over the ordinal logistic model due to the violation of the proportional odds assumption (ie, the hypothesis of parallel lines was rejected). Survey-weighted modified Poisson regression models,^18,19,20^ adjusted for the aforementioned confounders, were used to examine the associations between SDOH and the prevalence of CKM stages and to estimate prevalence ratios (PRs) and corresponding 95% CIs in all participants, in men only, and in women only. The χ^2^ automatic interaction detection classification tree was used for estimation of CKM stages or advanced vs nonadvanced CKM stages based on multiple SDOH. Sensitivity analyses were conducted using NHANES 2011-2018 data by replicating the main analyses for participants with advanced vs nonadvanced CKM stages.

As the PREVENT equations were not applicable to the imputed data, data imputation was not performed. Given the cross-sectional and exploratory nature of this study, which was designed to generate rather than test hypotheses, adjustments for multiple comparisons were not applied.^21,22,23^ Our results should be interpreted as exploratory due to the potential for type I error. A 2-sided *P* < .05 was considered statistically significant.

## Results

### Participant Characteristics

This study analyzed data from 29 722 participants, with a weighted mean (SE) age of 50.8 (0.1) years. Among them, a weighted 49.3% were female and 50.7% male, and 7.5% self-reported as Mexican American, 12.3% as non-Hispanic Black, 70.9% as non-Hispanic White, and 9.3% as other race and ethnicity. Participants with advanced CKM stages were more likely to be older, male, smokers, nondrinkers, and physically inactive and to have unfavorable SDOH (Table 1).

**Table 1.** Baseline Characteristics of Participants According to CKM Syndrome Stages, NHANES 1999-2018

Characteristic	Participants with CKM syndrome, No. (%)^a^						
	Stage 0	Stage 1	Stage 2	Stage 3	Stage 4	Nonadvanced stages (stages 0-2)	Advanced stages (stages 3 or 4)
No. of participants	3227	7991	13 223	2271	3010	24 441	5281
Age, mean (SE), y^b^	43.8 (0.3)	45.1 (0.2)	52.3 (0.2)	72.0 (0.2)	62.3 (0.3)	48.5 (0.1)	65.9 (0.2)
Age group, y							
30-44	1917 (58.1)	4276 (53.2)	3268 (27.2)	10 (0.5)	204 (8.5)	9461 (40.9)	214 (5.5)
45-64	1102 (37.0)	3147 (40.8)	7276 (56.5)	260 (11.2)	1190 (42.9)	11 525 (48.1)	1450 (31.0)
65-79	219 (4.9)	568 (6.0)	2679 (16.3)	2001 (88.4)	1616 (48.6)	3455 (11.0)	3617 (63.5)
Sex							
Female	1828 (60.0)	4035 (49.5)	6523 (48.0)	853 (41.3)	1246 (42.4)	12 386 (50.4)	2099 (42.0)
Male	1399 (40.0)	3956 (50.5)	6700 (52.0)	1418 (58.7)	1764 (57.6)	12 055 (49.6)	3182 (58.0)
Race and ethnicity							
Mexican American	379 (4.6)	1724 (10.0)	2545 (7.5)	384 (5.3)	421 (4.5)	4648 (7.9)	805 (4.8)
Non-Hispanic Black	732 (13.2)	1495 (12.5)	2531 (12.4)	325 (9.8)	426 (10.4)	4758 (12.6)	751 (10.2)
Non-Hispanic White	1711 (76.2)	3358 (68.3)	5463 (70.1)	1033 (73.7)	1527 (74.8)	10 532 (70.4)	2560 (74.4)
Other^c^	405 (6.0)	1414 (9.2)	2684 (10.0)	529 (11.2)	636 (10.4)	4503 (9.1)	1165 (10.7)
Education							
High school graduate or higher	2674 (89.2)	6070 (85.1)	9501 (82.6)	1418 (74.8)	1911 (74.4)	18 245 (84.5)	3329 (74.5)
Less than high school	552 (10.8)	1918 (14.9)	3710 (17.4)	848 (25.2)	1094 (25.6)	6180 (15.5)	1942 (25.5)
Marital status							
Married or living with a partner	2222 (72.2)	5538 (73.8)	8752 (70.9)	1388 (65.1)	1808 (66.2)	16 512 (72.1)	3196 (65.8)
Not married or living with a partner	963 (27.8)	2381 (26.2)	4343 (29.1)	866 (34.9)	1177 (33.8)	7687 (27.9)	2043 (34.2)
Family income to poverty ratio, %							
≥300	1551 (64.5)	3242 (57.6)	5140 (56.3)	600 (41.3)	802 (41.4)	12 473 (41.9)	3377 (58.6)
<300	1445 (35.5)	4077 (42.4)	6951 (43.7)	1436 (58.7)	1941 (58.6)	9933 (58.1)	1402 (41.4)
Food security							
Full security	2519 (86.8)	5678 (80.5)	9436 (80.9)	1732 (84.8)	2006 (75.4)	17 633 (81.7)	3738 (78.9)
Marginal, low, or very low security	639 (13.2)	2143 (19.5)	3488 (19.1)	496 (15.2)	948 (24.6)	6270 (18.3)	1444 (21.1)
Type of health insurance							
Private	2067 (73.1)	4764 (70.0)	7428 (67.6)	1047 (55.1)	1262 (51.8)	14 259 (69.3)	2309 (53.0)
Government or none	1147 (26.9)	3204 (30.0)	5756 (32.4)	1212 (44.9)	1736 (48.2)	10 107 (30.7)	2948 (47.0)
Employment status							
Employed, student, or retired	2697 (83.2)	6491 (84.6)	10238 (82.4)	1941 (87.7)	2048 (72.5)	19 326 (83.3)	3989 (78.2)
Unemployed	629 (16.8)	1495 (15.4)	2977 (17.6)	330 (12.3)	961 (27.5)	5101 (16.7)	1291 (21.8)
Home ownership							
Own home	2125 (74.4)	5096 (71.8)	9017 (76.0)	1709 (82.0)	2015 (74.4)	16 238 (74.3)	3724 (77.2)
Rent home or other arrangement	1061 (25.6)	2787 (28.2)	4020 (24.0)	528 (18.0)	958 (25.6)	7868 (25.7)	1486 (22.8)
Regular health care access							
At least 1 regular health care facility	2586 (82.5)	6359 (82.7)	11 461 (88.8)	2169 (96.1)	2875 (96.1)	20 406 (85.7)	5044 (96.1)
None or emergency department	641 (17.5)	1632 (17.3)	1762 (11.2)	102 (3.9)	135 (3.9)	4035 (14.3)	237 (3.9)
Alcohol consumption							
Nondrinker	717 (21.0)	2009 (24.7)	3768 (27.9)	755 (41.8)	945 (38.0)	6494 (25.7)	1700 (39.4)
Moderate drinker	250 (9.1)	634 (10.0)	1013 (10.0)	315 (18.9)	316 (15.0)	1897 (9.9)	631 (16.5)
Heavy drinker	1785 (69.9)	4114 (65.3)	6253 (10.1)	655 (39.3)	958 (47.0)	12 152 (64.4)	1613 (44.1)
Ever smoker							
Yes	1390 (43.0)	3374 (43.3)	6225 (48.8)	1342 (59.2)	1943 (66.2)	10 989 (46.0)	3285 (63.5)
No	1836 (57.0)	4610 (56.7)	6984 (51.2)	929 (40.8)	1067 (33.8)	13 430 (54.0)	1996 (36.5)
Physical activity							
Active	1632 (55.3)	3800 (50.7)	5358 (44.6)	734 (34.6)	972 (37.4)	10 790 (48.4)	1706 (36.4)
Inactive	1595 (44.7)	4191 (49.5)	7865 (55.4)	1537 (65.4)	2038 (62.6)	13 651 (51.6)	3575 (63.6)
Cumulative unfavorable SDOH							
<2	1613 (60.6)	3598 (56.1)	5870 (55.8)	850 (47.0)	1038 (44.6)	11 081 (56.6)	1888 (45.5)
≥2	1614 (39.4)	4393 (43.9)	7353 (44.2)	1421 (53.0)	1972 (55.4)	13 360 (43.4)	3393 (54.5)

Abbreviations: CKM, cardiovascular-kidney-metabolic; NHANES, National Health and Nutrition Examination Survey; SDOH, social determinants of health.

^a^ Weighted percentages; differences in baseline characteristics across the 5 CKM syndrome stages and between advanced and nonadvanced CKM syndrome stages were assessed using survey-weighted linear regression for continuous variables and survey-weighted Pearson χ2 tests for categorical variables (all *P* < .001).

^b^ Weighted.

^c^ Includes other Hispanic or other race, including multiracial and any race other than Black or White.

### Prevalence of CKM Stages by SDOH

The age-standardized prevalence of CKM stages 0, 1, 2, 3, and 4 was 13.6% (95% CI, 13.0%-14.3%), 29.9% (95% CI, 29.1%-30.7%), 43.7% (95% CI, 42.9%-44.5%), 4.7% (95% CI, 4.4%-5.0%), and 8.1% (95% CI, 7.6%-8.5%), respectively (Table 2). Significant differences in the prevalence of CKM stages were observed across age groups, sexes, and SDOH.

**Table 2.** Prevalence of CKM Syndrome Stages by Age, Sex, and SDOH, NHANES 1999-2018

Characteristic	CKM syndrome, % (95% CI)^a^				
	Stage 0	Stage 1	Stage 2	Stage 3	Stage 4
No. of participants	3227	7991	13 223	2271	3010
Total	13.6 (13.0-14.3)	29.9 (29.1-30.7)	43.7 (42.9-44.5)	4.7 (4.4-5.0)	8.1 (7.6-8.5)
Age group, y					
30-44	21.8 (20.6-22.9)	43.7 (42.3-45.0)	32.6 (31.4-33.8)	0.1 (0.0-0.1)	1.9 (1.6-2.3)
45-64	11.0 (10.1-11.9)	26.5 (25.4-27.7)	53.7 (52.4-55.0)	1.2 (1.0-1.4)	7.7 (7.1-8.3)
65-79	3.7 (3.2-4.3)	10.0 (8.8-11.4)	39.8 (38.3-41.3)	24.3 (22.9-25.7)	22.3 (21.0-23.6)
Sex					
Male	10.5 (9.8-11.3)	29.2 (28.1-30.3)	44.8 (43.9-45.8)	5.9 (5.5-6.3)	9.6 (8.9-10.2)
Female	17.0 (16.1-17.9)	30.6 (29.7-31.6)	42.1 (41.1-43.1)	3.6 (3.3-4.0)	6.7 (6.2-7.2)
Race and ethnicity					
Mexican American	7.0 (6.2-7.9)	34.2 (32.8-35.7)	46.1 (44.6-47.5)	6.0 (5.4-6.7)	6.6 (5.9-7.4)
Non-Hispanic Black	8.2 (7.4-9.2)	27.9 (26.8-29.1)	47.1 (46.0-48.3)	6.9 (6.5-7.4)	9.8 (9.1-10.5)
Non-Hispanic White	15.3 (14.4-16.1)	29.7 (28.7-30.7)	42.7 (41.7-43.8)	4.4 (4.1-4.7)	8.0 (7.4-8.5)
Other^b^	13.4 (12.3-14.6)	28.4 (26.7-30.1)	45.2 (43.5-47.0)	5.0 (4.4-5.7)	8.0 (6.9-9.3)
Education					
High school graduate or higher	14.5 (13.8-15.2)	30.4 (29.5-31.2)	43.3 (42.4-44.2)	4.4 (4.1-4.8)	7.4 (6.9-7.8)
Less than high school	9.0 (8.1-10.0)	27.5 (26.1-29.0)	46.1 (44.6-47.6)	5.8 (5.3-6.3)	11.5 (10.6-12.5)
Marital status					
Married or living with a partner	13.6 (12.9-14.4)	30.7 (29.8-31.6)	43.4 (42.5-44.4)	4.5 (4.2-4.9)	7.7 (7.2-8.2)
Not married or living with a partner	13.4 (12.4-14.4)	27.9 (26.6-29.2)	44.5 (43.1-46.0)	5.1 (4.7-5.5)	9.2 (8.5-9.9)
Family income to poverty ratio, %					
≥300	15.9 (15.0-16.9)	30.8 (29.6-32.0)	43.2 (42.0-44.4)	3.9 (3.6-4.3)	6.2 (5.7-6.7)
<300	10.7 (10.0-11.5)	28.3 (27.3-29.4)	44.9 (43.8-45.9)	5.5 (5.1-5.9)	10.6 (10.0-11.3)
Food security					
Full security	14.9 (14.1-15.7)	30.2 (29.3-31.1)	43.2 (42.3-44.1)	4.6 (4.3-4.9)	7.1 (6.7-7.6)
Marginal, low, or very low security	8.6 (7.8-9.5)	28.1 (26.7-29.4)	45.0 (43.6-46.5)	5.7 (5.1-6.4)	12.6 (11.6-13.7)
Type of health insurance					
Private	14.6 (13.8-15.5)	30.8 (29.9-31.7)	43.5 (42.5-44.6)	4.5 (4.1-4.8)	6.6 (6.1-7.1)
Government or none	11.3 (10.5-12.2)	28.0 (26.8-29.2)	44.1 (43.0-45.3)	5.2 (4.8-5.6)	11.4 (10.7-12.1)
Employment status					
Employed, student, or retired	13.9 (13.2-14.6)	30.9 (30.0-31.9)	43.7 (42.9-44.6)	4.7 (4.4-4.9)	6.8 (6.4-7.2)
Unemployed	12.5 (11.3-13.8)	25.0 (23.8-26.4)	43.6 (42.0-45.3)	4.8 (4.2-5.6)	14.0 (12.9-15.2)
Home ownership					
Own home	14.4 (13.7-15.2)	30.1 (29.2-31.0)	43.6 (42.7-44.5)	4.6 (4.3-4.9)	7.4 (6.9-7.8)
Rent home or other arrangement	11.7 (10.8-12.5)	29.0 (27.7-30.3)	43.3 (42.0-44.6)	5.4 (4.9-6.0)	10.6 (9.9-11.4)
Regular health care access					
At least 1 regular health care facility	13.3 (12.7-14.1)	29.2 (28.3-30.0)	44.3 (43.4-45.2)	4.7 (4.5-5.0)	8.5 (8.0-8.9)
None or emergency department	15.5 (14.0-17.0)	35.2 (33.3-37.3)	41.7 (39.6-43.8)	4.1 (3.2-5.1)	3.6 (2.9-4.4)
Cumulative unfavorable SDOH					
<2	15.4 (14.5-16.4)	30.9 (30.0-30.7)	43.2 (42.1-44.3)	4.1 (3.8-4.5)	6.4 (5.9-6.9)
≥2	11.4 (10.7-12.2)	28.5 (27.4-29.6)	44.3 (42.2-45.4)	5.5 (5.1-5.8)	10.3 (9.7-11.0)

Abbreviations: CKM, cardiovascular-kidney-metabolic; NHANES, National Health and Nutrition Examination Survey; SDOH, social determinants of health.

^a^ Data are presented as age-standardized percentages (95% CIs) except for age groups. All estimates accounted for complex survey design (as in Table 3). The revalence of CKM syndrome stages was calculated using the direct standardization method, standardized to the 2010 US census population with 3 age categories of 30 to 44 years, 45 to 64 years, and 65 to 79 years. Differences in the prevalence of CKM syndrome stages across age groups, sexes, races and ethnicities, and SDOH were determined using survey-weighted and age-standardized Pearson χ^2^ tests (all *P* < .001).

^b^ Includes other Hispanic or other race, including multiracial and any race other than Black or White.

Participants with a lower education level had significantly higher RRRs for CKM stage 2 (1.38 [95% CI, 1.18-1.61]), stage 3 (1.44 [95% CI, 1.12-1.85]), and stage 4 (1.97 [95% CI, 1.63-2.38]) compared with those with a higher education level (Table 3). Similar trends were seen in family income to poverty ratio (<300% vs ≥300%), food insecurity vs full food security, unemployment vs employment, and without vs with home ownership. In contrast, participants with no regular care or only emergency department access had lower RRRs for advanced CKM stages (stage 3: 0.61 [95% CI, 0.40-0.94]; stage 4: 0.32 [95% CI, 0.23-0.46]) compared with those with access to at least 1 regular health care facility.

**Table 3.** Relative Risk Ratio of CKM Syndrome Stages by SDOH, NHANES 1999-2018

Characteristic	RRR (95% CI) of CKM syndrome^a^			
	Stage 1	Stage 2	Stage 3	Stage 4
Education				
High school graduate or higher	1 [Reference]	1 [Reference]	1 [Reference]	1 [Reference]
Less than high school	1.13 (0.96-1.34)	1.38 (1.18-1.61)	1.44 (1.12-1.85)	1.97 (1.63-2.38)
Marital status				
Married or living with a partner	1 [Reference]	1 [Reference]	1 [Reference]	1 [Reference]
Not married or living with a partner	0.93 (0.82-1.04)	1.04 (0.92-1.19)	1.13 (0.89-1.42)	1.23 (1.05-1.44)
Family income to poverty ratio, %				
<300	1 [Reference]	1 [Reference]	1 [Reference]	1 [Reference]
≥300	1.25 (1.10-1.43)	1.41 (1.23-1.61)	1.99 (1.60-2.49)	2.25 (1.89-2.67)
Food security				
Full security	1 [Reference]	1 [Reference]	1 [Reference]	1 [Reference]
Marginal, low, or very low security	1.41 (1.23-1.62)	1.67 (1.44-1.94)	2.55 (1.91-3.39)	3.29 (2.67-4.04)
Type of health insurance				
Private	1 [Reference]	1 [Reference]	1 [Reference]	1 [Reference]
Government or none	1.00 (0.88-1.14)	1.10 (0.97-1.25)	1.36 (1.10-1.68)	1.76 (1.50-2.07)
Employment status				
Employed, student, or retired	1 [Reference]	1 [Reference]	1 [Reference]	1 [Reference]
Unemployed	0.91 (0.79-1.06)	1.22 (1.04-1.42)	2.08 (1.60-2.72)	2.98 (2.41-3.68)
Home ownership				
Own home	1 [Reference]	1 [Reference]	1 [Reference]	1 [Reference]
Rent home or other arrangement	1.12 (0.99-1.27)	1.20 (1.08-1.34)	1.67 (1.33-2.10)	1.85 (1.59-2.16)
Regular health care access				
At least 1 regular health care facility	1 [Reference]	1 [Reference]	1 [Reference]	1 [Reference]
None or emergency department	0.90 (0.79-1.03)	0.69 (0.60-0.80)	0.61 (0.40-0.94)	0.32 (0.23-0.46)
Cumulative unfavorable SDOH				
<2	1 [Reference]	1 [Reference]	1 [Reference]	1 [Reference]
≥2	1.14 (1.01-1.29)	1.34 (1.19-1.51)	1.95 (1.58-2.41)	2.10 (1.76-2.51)

Abbreviations: CKM, cardiovascular-kidney-metabolic; NHANES, National Health and Nutrition Examination Survey; RRR, relative risk ratio; SDOH, social determinants of health.

^a^ All estimates accounted for complex survey designs. Multinominal logistic regression models adjusted for baseline age, sex, race and ethnicity, alcohol consumption, smoking status, and physical activity were used to estimate RRRs and 95% CIs for associations between SDOHs and the prevalence of CKM stages.

### Prevalence of Advanced CKM Stages by SDOH

**Figure 1.**
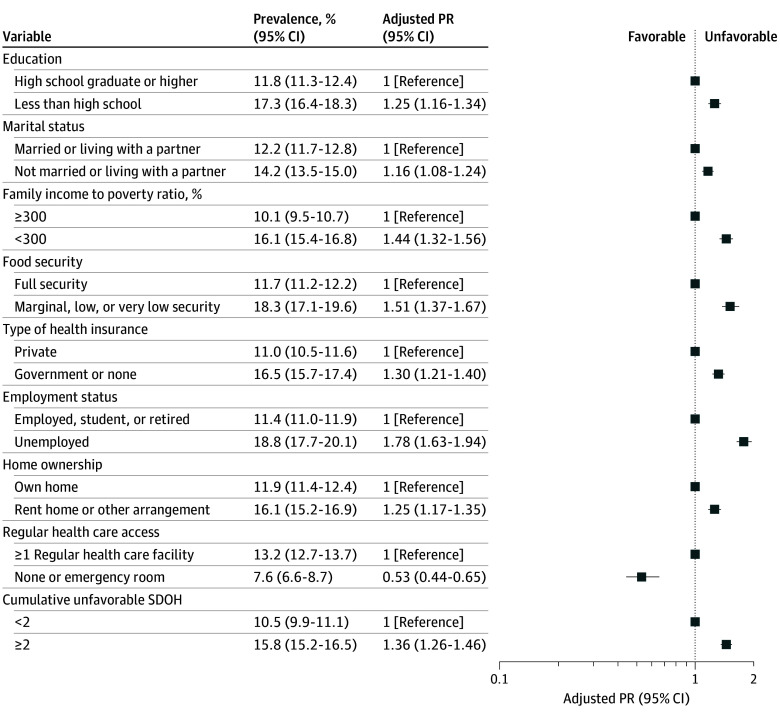
Prevalence of Advanced Cardiovascular-Kidney-Metabolic Syndrome Stages by Social Determinants of Health (SDOH), National Health and Nutrition Examination Survey 1999-2018 Survey-weighted modified Poisson regression models adjusted for baseline age, sex, race and ethnicity, alcohol consumption, smoking status, and physical activity were used (all *P* < .001). PR indicates prevalence ratio.

A higher prevalence of advanced CKM stages was observed among participants, compared with their counterparts, who were unmarried or not living with a partner (age-standardized prevalence, 14.2% [95% CI, 13.5%-15.0%] vs 12.2% [95% CI, 11.7%-12.8%]), had a family income to poverty ratio less than 300% (16.1% [95% CI, 15.4%-16.8%] vs 10.1% [95% CI, 9.5%-10.7%]), had food insecurity (18.3% [95% CI, 17.1%-19.6%] vs 11.7% [95% CI, 11.2%-12.2%]), had government health insurance or lacked health insurance (16.5% [95% CI, 15.7%-17.4%] vs 11.0% [95% CI, 10.5%-11.6%]), were unemployed (18.8% [95% CI, 17.7%-20.1%] vs 11.4% [95% CI, 11.0%-11.9%]), had a lower education level (17.3% [95% CI, 16.4%-18.3%] vs 11.8% [95% CI, 11.3%-12.4%]), and lived in a rented home (16.1% [95% CI, 15.2%-16.9%] vs 11.9% [95% CI, 11.4%-12.4%]) (Figure 1). Participants with 2 or more cumulative unfavorable SDOH had a higher prevalence of advanced CKM stages (15.8% [95% CI, 15.2%-16.5%] vs 10.5% [95% CI, 9.9%-11.1%] with <2 unfavorable SDOH; adjusted PR, 1.36 [95% CI, 1.26-1.46]). In contrast, participants with no regular health care or who used emergency departments were less likely to have advanced CKM stages compared with those with access to at least 1 regular health care facility (7.6% [95% CI, 6.6%-8.7%] vs (13.2% [95% CI, 12.7%-13.7%]). These findings remained robust in the sensitivity analyses except for education (eFigure 2 in Supplement 1). Decision trees indicated that employment status, health insurance, and family income to poverty ratio were key SDOH influencing CKM stage risk (eFigure 3 in Supplement 1).

**Figure 2.**
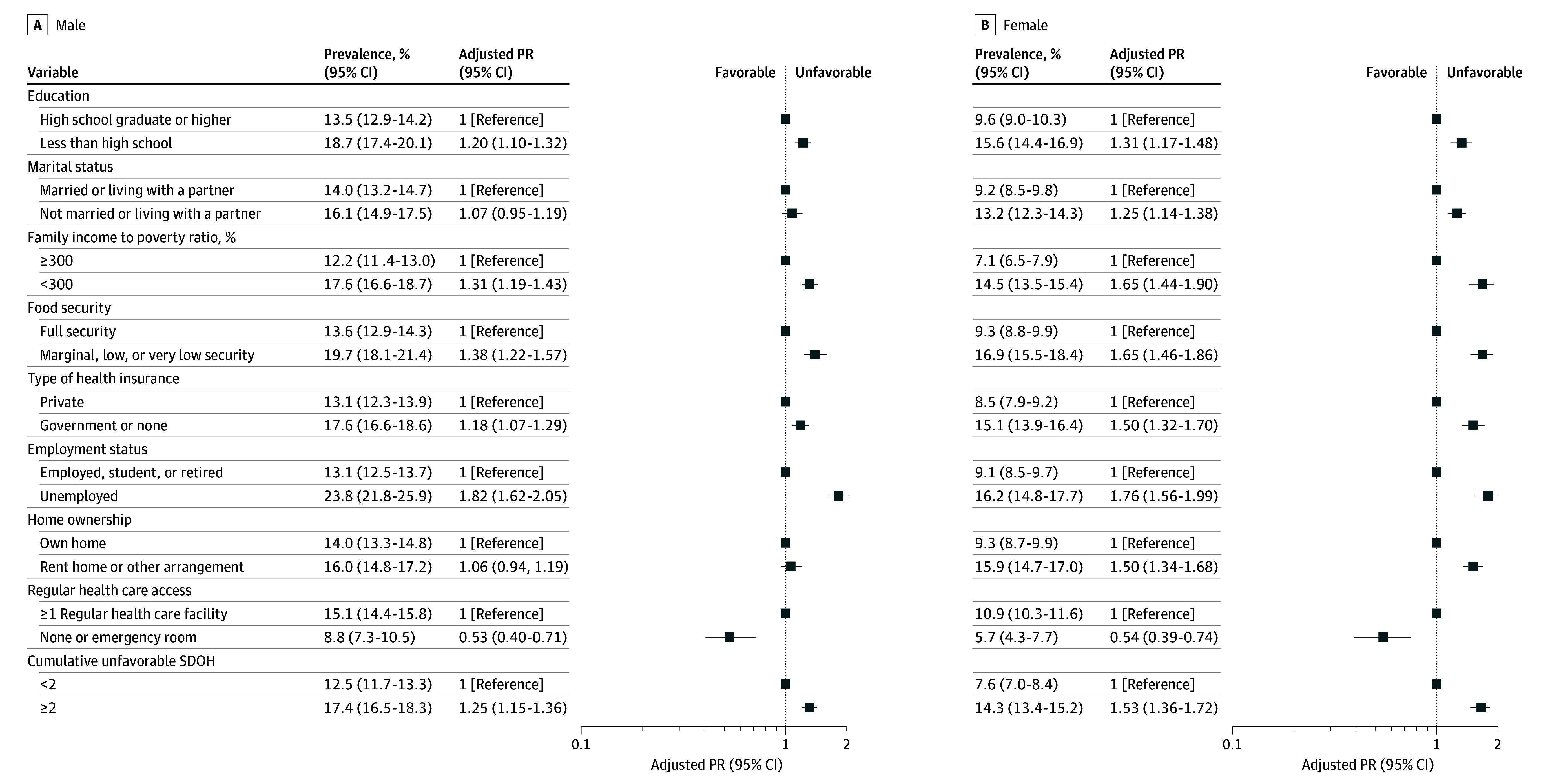
Prevalence of Advanced Cardiovascular-Kidney-Metabolic Syndrome Stages by Social Determinants of Health (SDOH) Among Males and Females, National Health and Nutrition Examination Survey 1999-2018 Survey-weighted modified Poisson regression models adjusted for baseline age, race and ethnicity, alcohol consumption, smoking status, and physical activity were used (all *P* < .001). PR indicates prevalence ratio.

Both men and women with unfavorable education, family income to poverty ratio, food security, health insurance, and employment status were more likely to have a higher prevalence of advanced CKM stages (Figure 2). Among women, those who were unmarried or not living with a partner (13.2% [95% CI, 12.3%-14.3%] vs 9.2% [95% CI, 8.5%-9.8%] married or living with a partner; adjusted PR, 1.25 [95% CI, 1.14-1.38]) or without home ownership (15.9% [95% CI, 14.7%-17.0%] vs 9.3% [95% CI, 8.7%-9.9%] owning their home; adjusted PR, 1.50 [95% CI, 1.34-1.68]) were more likely to have advanced CKM stages compared with their married or home-owning counterparts, respectively. All results remained robust in the sensitivity analyses except for education (eFigure 4 in Supplement 1). Decision trees highlighted employment status and family income to poverty ratio as key SDOH associated with advanced CKM stages in both the main and sensitivity analyses (eFigures 5 and 6 in Supplement 1).

## Discussion

This cross-sectional study of a nationally representative sample of US adults showed that individuals with unfavorable SDOH, especially low family income, food insecurity, or unemployment, had a significantly higher likelihood of CKM stages 2 to 4 or advanced CKM stages compared with those with favorable SDOH. Notably, women who were unmarried or not living with a partner or living in a rented home were more likely to have advanced CKM stages, whereas these results were not observed in men. All findings except education remained robust in the sensitivity analyses.

Previous research has extensively documented the association of adverse SDOH with the prevalence and risks of major CKM components, including obesity, diabetes, CVD, and CKD.^24,25,26,27,28^ In one study, unfavorable SDOH, such as food insecurity, were adversely associated with diabetes self-care behaviors, including checking blood glucose and eating vegetables.^29^ In other studies, social disadvantages were associated with more prevalent CVD risk factors and incident CVD.^26,27^ Adverse associations of SDOH with CKD have also been identified.^28^ Our findings aligned with these and several other previous studies.^30,31,32,33,34,35,36^ Moreover, our study not only confirms that adults with unfavorable SDOH, such as low family income to poverty ratio and food insecurity, were more likely to have CKM stages 2 to 4 and advanced CKM stages but also underscores the need for targeted screening, prevention, and treatment efforts for unemployed adults and those with low income or food insecurity. Unexpectedly, we found that advanced CKM stages were more common in adults who access at least 1 regular health care facility than those without a routine place for health care. This finding contradicts previous studies, possibly due to reverse causality inherent in cross-sectional studies, as individuals with more severe disease may be more likely to seek regular health care services.

In this analysis, we found that adults with 2 or more unfavorable SDOH or with specific unfavorable SDOH (ie, low family income to poverty ratio, food insecurity, government health insurance or lack of health insurance, and unemployment) were more likely to have advanced CKM stages compared with those with fewer than 2 unfavorable SDOHs. These associations differed by sex. Notably, women who were unmarried or not living with a partner or who did not own their home were more likely to have advanced CKM stages, while such associations were not observed in men. Previous studies have highlighted the importance of housing factors in cardiometabolic health and overall well-being.^37,38,39^ For instance, lack of home ownership is a key housing factor associated with not only poor physical health outcomes but also mental health outcomes.^37,40,41^ Moreover, recent studies have indicated that living in a privately rented home was associated with accelerated biological aging, highlighting the substantial health implications of home ownership.^42^ However, few studies have explored sex differences in relation to home ownership and CVD or metabolic disease, warranting further investigation in future well-designed studies.

Our findings highlighted important disparities in the prevalence of CKM syndrome across SDOH and sex, emphasizing the importance of screening for and addressing unfavorable SDOHs in the prevention and treatment of CKM syndrome. Specifically, key SDOH, such as low family income, food insecurity, and unemployment, should be prioritized for intervention across the population, while housing factors, such as living in a rented home and not living with a partner, deserve special attention in women. In our study, decision trees were used to estimate which CKM stages and advanced CKM stages are associated with SDOH, identifying employment status and family income as crucial factors. Existing SDOH screening tools, such as Health Leads, IHELP, and WE CARE,^1,43,44^ should be evaluated for their effectiveness in identifying critical factors, such as employment status and family income, in the context of CKM syndrome.

### Limitations

This study has several limitations. First, the cross-sectional design limited our ability to establish causal relationships between SDOH and CKM stages. Second, some CVD-related data that were used to define advanced CKM stages, such as cardiac biomarkers, echocardiography, and coronary angiography, were not available in the NHANES database, potentially leading to an underestimation of advanced CKM stages (stages 3 or 4). Third, certain SDOHs, such as social cohesion, structural racism, and neighborhood and community environments, were not assessed due to data limitations. Finally, the applicability of the PREVENT equations was limited to adults aged 30 to 79 years and those without extreme values of CVD risk factors, which may affect the generalizability of our findings.

## Conclusions

In this cross-sectional study of a nationally representative sample of US adults, we found statistically significant disparities in the prevalence of CKM stages by SDOH, with notable sex differences. Our findings underscore the disproportionate burden of CKM syndrome among adults with unfavorable SDOH and suggest that addressing these adverse factors might be crucial for the prevention and treatment of CKM syndrome. Specifically, unemployment, low family income, and food insecurity in all adults require targeted attention. Additionally, female individuals who are unmarried or living in a rented home should receive particular attention. Given the limitations, our findings should be interpreted with caution and validated by future studies.

## Notes

Supplement 1. **eFigure 1.** Flow Chart of the Study Population **eFigure 2.** Prevalence (95% CI) of Advanced Cardiovascular-Kidney-Metabolic Syndrome Stages by Social Determinants of Health, NHANES 2011-2018 **eFigure 3.** Decision Tree Created Using the Chi-Squared Automatic Interaction Detection Model for Cardiovascular-Kidney-Metabolic Syndrome Stages, NHANES 1999-2018 **eFigure 4.** Prevalence (95% CI) of Advanced Cardiovascular-Kidney-Metabolic Syndrome Stages by Social Determinants of Health Among Males and Females, NHANES 2011-2018 **eFigure 5.** Decision Tree Created Using the Chi-Squared Automatic Interaction Detection Model for Advanced vs Nonadvanced Cardiovascular-Kidney-Metabolic Syndrome Stages, NHANES 1999-2018 **eFigure 6.** Decision Tree Created Using the Chi-Squared Automatic Interaction Detection Model for Advanced vs Nonadvanced Cardiovascular-Kidney-Metabolic Syndrome Stages, NHANES 2011-2018

Supplement 2.
Data Sharing Statement
