# Supplementary material for: Prevalence of Cardiovascular-Kidney-Metabolic Syndrome Stages by Social Determinants of Health
Source: JAMA Netw Open. 2024 Nov 18;7(11):e2445309. doi: 10.1001/jamanetworkopen.2024.45309 (PMC11574692; doi:10.1001/jamanetworkopen.2024.45309)
Supplement: Supplement 1. — eFigure 1. Flow Chart of the Study Population eFigure 2. Prevalence (95% CI) of Advanced Cardiovascular-Kidney-Metabolic Syndrome Stages by Social Determinants of Health, NHANES 2011-2018 eFigure 3. Decision Tree Created Using the Chi-Squared Automatic Interaction Detection Model for Cardiovascular-Kidney-Metabolic Syndrome Stages, NHANES 1999-2018 eFigure 4. Prevalence (95% CI) of Advanced Cardiovascular-Kidney-Metabolic Syndrome Stages by Social Determinants of Health Among Males and Females, NHANES 2011-2018 eFigure 5. Decision Tree Created Using the Chi-Squared Automatic Interaction Detection Model for Advanced vs Nonadvanced Cardiovascular-Kidney-Metabolic Syndrome Stages, NHANES 1999-2018 eFigure 6. Decision Tree Created Using the Chi-Squared Automatic Interaction Detection Model for Advanced vs Nonadvanced Cardiovascular-Kidney-Metabolic Syndrome Stages, NHANES 2011-2018 [file jamanetwopen-e2445309-s001.pdf]

Supplemental Online Content

Zhu R, Wang R, He J, et al. Prevalence of cardiovascular-kidney-metabolic syndrome stages by social determinants of health. *JAMA Netw Open*. 2024;7(11):e2445309. doi:10.1001/jamanetworkopen.2024.45309

eFigure 1. Flow Chart of the Study Population

eFigure 2. Prevalence (95% CI) of Advanced Cardiovascular-Kidney-Metabolic Syndrome Stages by Social Determinants of Health, NHANES 2011-2018

eFigure 3. Decision Tree Created Using the Chi-Squared Automatic Interaction Detection Model for Cardiovascular-Kidney-Metabolic Syndrome Stages, NHANES 1999-2018

eFigure 4. Prevalence (95% CI) of Advanced Cardiovascular-Kidney-Metabolic Syndrome Stages by Social Determinants of Health Among Males and Females, NHANES 2011-2018

eFigure 5. Decision Tree Created Using the Chi-Squared Automatic Interaction Detection Model for Advanced vs Nonadvanced Cardiovascular-Kidney-Metabolic Syndrome Stages, NHANES 1999-2018

eFigure 6. Decision Tree Created Using the Chi-Squared Automatic Interaction Detection Model for Advanced vs Nonadvanced Cardiovascular-Kidney-Metabolic Syndrome Stages, NHANES 2011-2018

This supplemental material has been provided by the authors to give readers additional information about their work.

© 2024 Zhu R et al. *JAMA Network Open.*

eFigure 1. Flow Chart of the Study Population

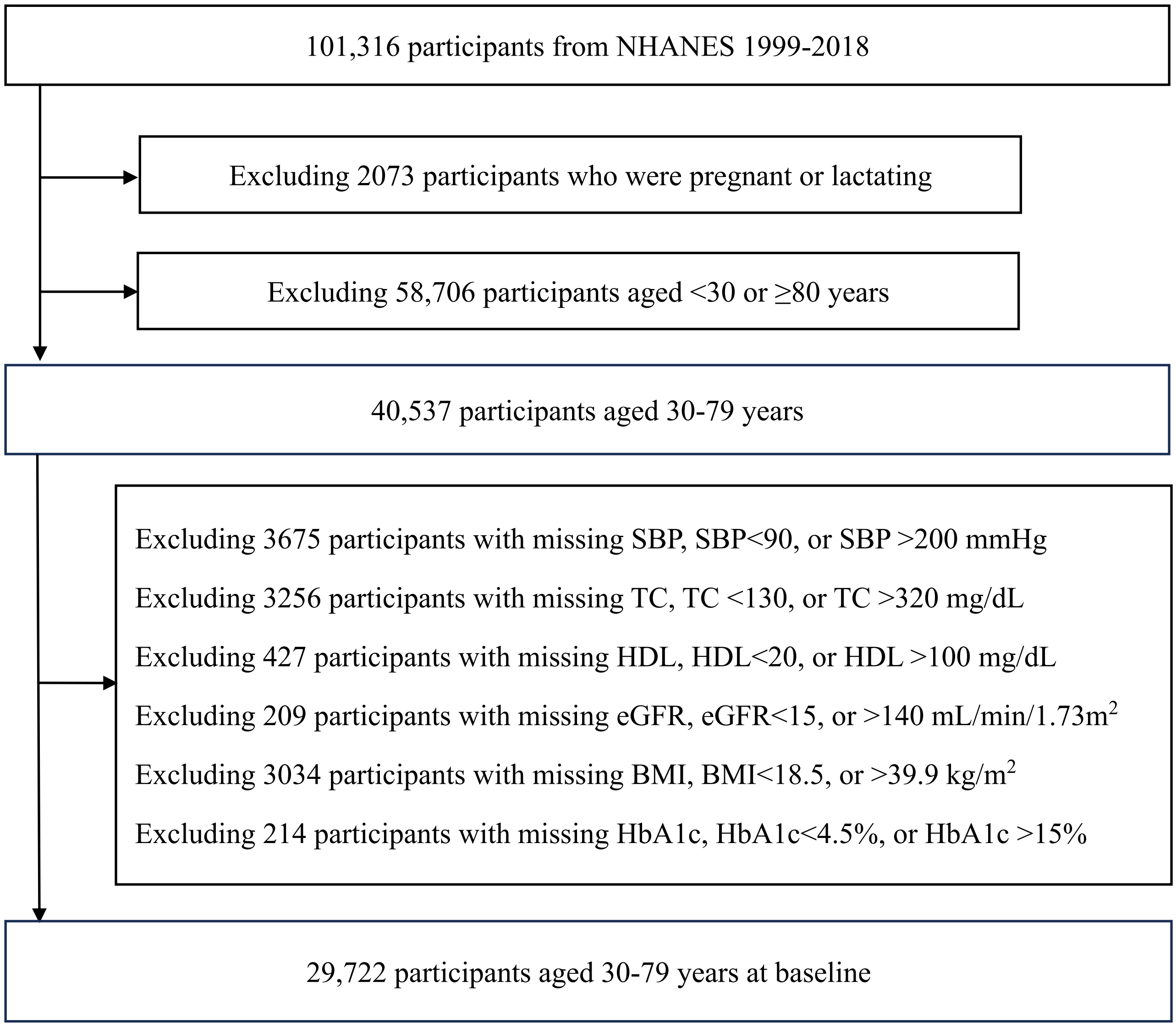

Abbreviations: BMI, body mass index; eGFR, estimated glomerular filtration rate; HbA1c, glycosylated hemoglobin A1c; HDL, high-density lipoprotein cholesterol; SBP, systolic blood pressure; TC, total cholesterol.

© 2024 Zhu R et al. *JAMA Network Open.*

eFigure 2. Prevalence (95% CI) of Advanced Cardiovascular-Kidney-Metabolic Syndrome Stages by Social Determinants of Health, NHANES 2011-2018

	Prevalence, % (95% CI)	Prevalence ratio (95% CI)	*P* value
**Education**			.10
High school graduate or higher	12.1 (11.2, 13.1)	1.00 (Reference)	
Less than high school	16.2 (14.7, 17.7)	1.12 (0.98, 1.28)	
**Marital status**			<.001
Married or living with a partner	11.9 (11.0, 12.8)	1.00 (Reference)	
Not married nor living with a partner	14.7 (13.4, 16.1)	1.19 (1.08, 1.31)	
**Family income-to-poverty ratio**			<.001
≥300%	9.5 (8.7, 10.5)	1.00 (Reference)	
<300%	16.6 (15.4, 17.9)	1.52 (1.34, 1.74)	
**Food security**			<.001
Full security	11.2 (10.4, 11.9)	1.00 (Reference)	
Marginal, low, or very low security	18.6 (16.9, 20.6)	1.60 (1.37, 1.85)	
**Type of health insurance**			<.001
Private	10.3 (9.4, 11.3)	1.00 (Reference)	
Government or none	17.2 (15.9, 18.5)	1.42 (1.26, 1.59)	
**Employment status**			<.001
Employed, student, or retired	11.3 (10.5, 12.0)	1.00 (Reference)	
Unemployed	19.2 (17.3, 21.2)	1.81 (1.55, 2.10)	
**Home ownership**			<.001
Own home	11.5 (10.8, 12.3)	1.00 (Reference)	
Rent home or other arrangement	16.6 (15.3, 18.0)	1.31 (1.17, 1.46)	
**Regular health-care access**			<.001
At least one regular health-care facility	13.1 (12.3, 14.0)	1.00 (Reference)	
None or emergency room	7.9 (6.5, 9.5)	0.53 (0.39, 0.71)	
**Cumulative unfavorable SDOHs**			<.001
<2	9.9 (9.0, 11.0)	1.00 (Reference)	
≥2	16.0 (15.0, 17.1)	1.47 (1.31, 1.64)	

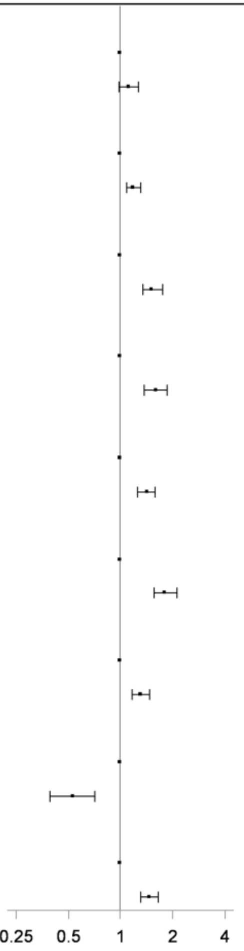

Abbreviations: CI, confidence interval; SDOHs, social determinants of health. Survey-weighted modified Poisson regression models adjusted for baseline age, sex, race/ethnicity, alcohol consumption, smoking status, and physical activity were used.

© 2024 Zhu R et al. *JAMA Network Open.*

eFigure 3. Decision Tree Created Using the Chi-Squared Automatic Interaction Detection Model for Cardiovascular-Kidney-Metabolic Syndrome Stages, NHANES 1999-2018

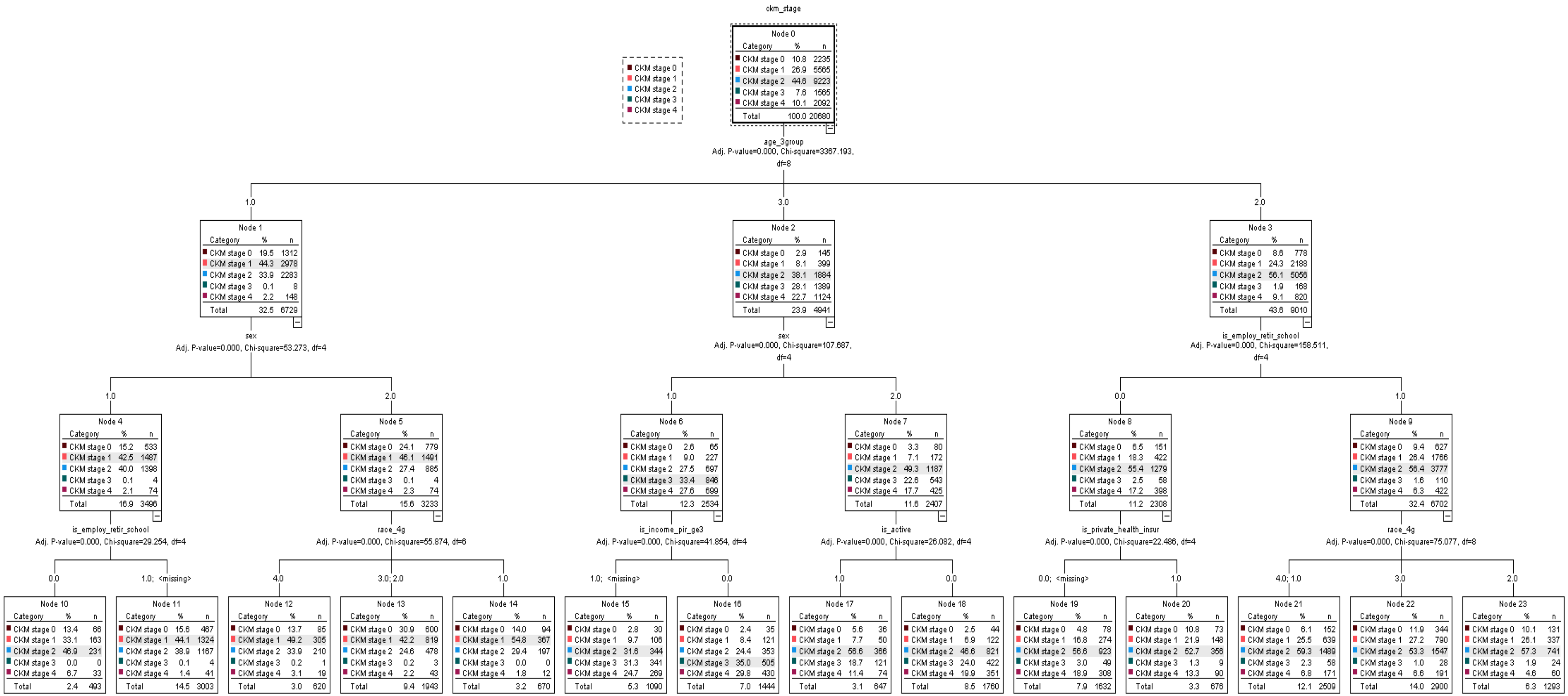

Abbreviation: CKM, cardiovascular-kidney-metabolic syndrome.

© 2024 Zhu R et al. *JAMA Network Open.*

eFigure 4. Prevalence (95% CI) of Advanced Cardiovascular-Kidney-Metabolic Syndrome Stages by Social Determinants of Health Among Males and Females, NHANES 2011-2018

	Male			Female		
	Prevalence, % (95% CI)	Prevalence ratio, (95% CI)	*P* value	Prevalence, % (95% CI)	Prevalence ratio, (95% CI)	*P* value
**Education**			.26			.11
High school graduate or higher	13.9 (12.8, 15.0)	1.00 (Reference)		10.0 (8.9, 11.2)	1.00 (Reference)	
Less than high school	17.4 (15.4, 19.5)	1.09 (0.94, 1.26)		14.4 (12.7, 16.3)	1.16 (0.96, 1.41)	
**Marital status**			.15			.002
Married or living with a partner	13.5 (12.5, 14.7)	1.00 (Reference)		8.9 (7.9, 10.0)	1.00 (Reference)	
Not married nor living with a partner	17.0 (14.8, 19.5)	1.13 (0.96, 1.32)		13.5 (12.0, 15.2)	1.27 (1.10, 1.47)	
**Family income-to-poverty ratio**			<.001			<.001
≥300%	11.6 (10.5, 12.9)	1.00 (Reference)		6.7 (5.8, 7.9)	1.00 (Reference)	
<300%	18.0 (16.3, 19.8)	1.36 (1.18, 1.56)		15.1 (13.6, 16.7)	1.77 (1.41, 2.23)	
**Food security**			.001			<.001
Full security	13.1 (12.1, 14.3)	1.00 (Reference)		8.7 (7.8, 9.6)	1.00 (Reference)	
Marginal, low, or very low security	19.6 (17.3, 22.0)	1.40 (1.15, 1.69)		17.6 (15.6, 19.8)	1.83 (1.54, 2.17)	
**Type of health insurance**			.02			<.001
Private	12.6 (11.4, 14.0)	1.00 (Reference)		7.6 (6.6, 8.8)	1.00 (Reference)	
Government or none	18.0 (16.3, 19.8)	1.21 (1.03, 1.41)		16.0 (14.1, 18.1)	1.76 (1.43, 2.17)	
**Employment status**			<.001			<.001
Employed, student, or retired	13.0 (12.1, 14.0)	1.00 (Reference)		8.9 (8.0, 9.9)	1.00 (Reference)	
Unemployed	23.4 (20.4, 26.7)	1.78 (1.50, 2.12)		16.8 (14.6, 19.3)	1.85 (1.51, 2.26)	
**Home ownership**			.20			<.001
Own home	13.7 (12.6, 14.8)	1.00 (Reference)		9.0 (8.0, 10.0)	1.00 (Reference)	
Rent home or other arrangement	16.4 (14.5, 18.6)	1.13 (0.94, 1.37)		16.5 (14.8, 18.3)	1.55 (1.28, 1.87)	
**Regular health-care access**			.003			.01
At least one regular health-care facility	15.1 (14.0, 16.1)	1.00 (Reference)		10.9 (9.9, 12.0)	1.00 (Reference)	
None or emergency room	8.5 (6.4, 11.4)	0.53 (0.35, 0.81)		6.7 (4.4, 10.1)	0.53 (0.32, 0.87)	
**Cumulative unfavorable SDOHs**			<.001			<.001
<2	11.9 (10.8, 13.1)	1.00 (Reference)		7.1 (6.0, 8.4)	1.00 (Reference)	
≥2	17.7 (16.2, 19.3)	1.39 (1.22, 1.58)		14.4 (13.0, 15.8)	1.62 (1.35, 1.94)	

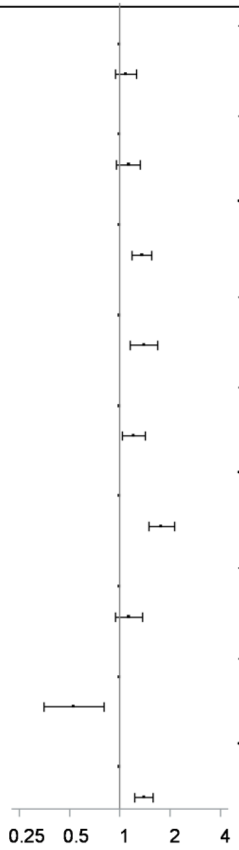

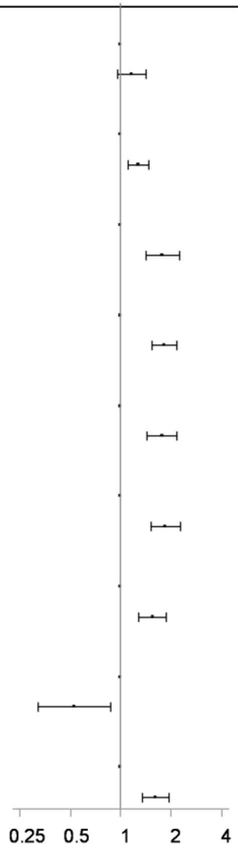

Abbreviations: CI, confidence interval; SDOHs, social determinants of health. Survey-weighted modified Poisson regression models adjusted for baseline age, race/ethnicity, alcohol consumption, smoking status, and physical activity were used.

© 2024 Zhu R et al. *JAMA Network Open.*

eFigure 5. Decision Tree Created Using the Chi-Squared Automatic Interaction Detection Model for Advanced vs Nonadvanced Cardiovascular-Kidney-Metabolic Syndrome Stages, NHANES 1999-2018

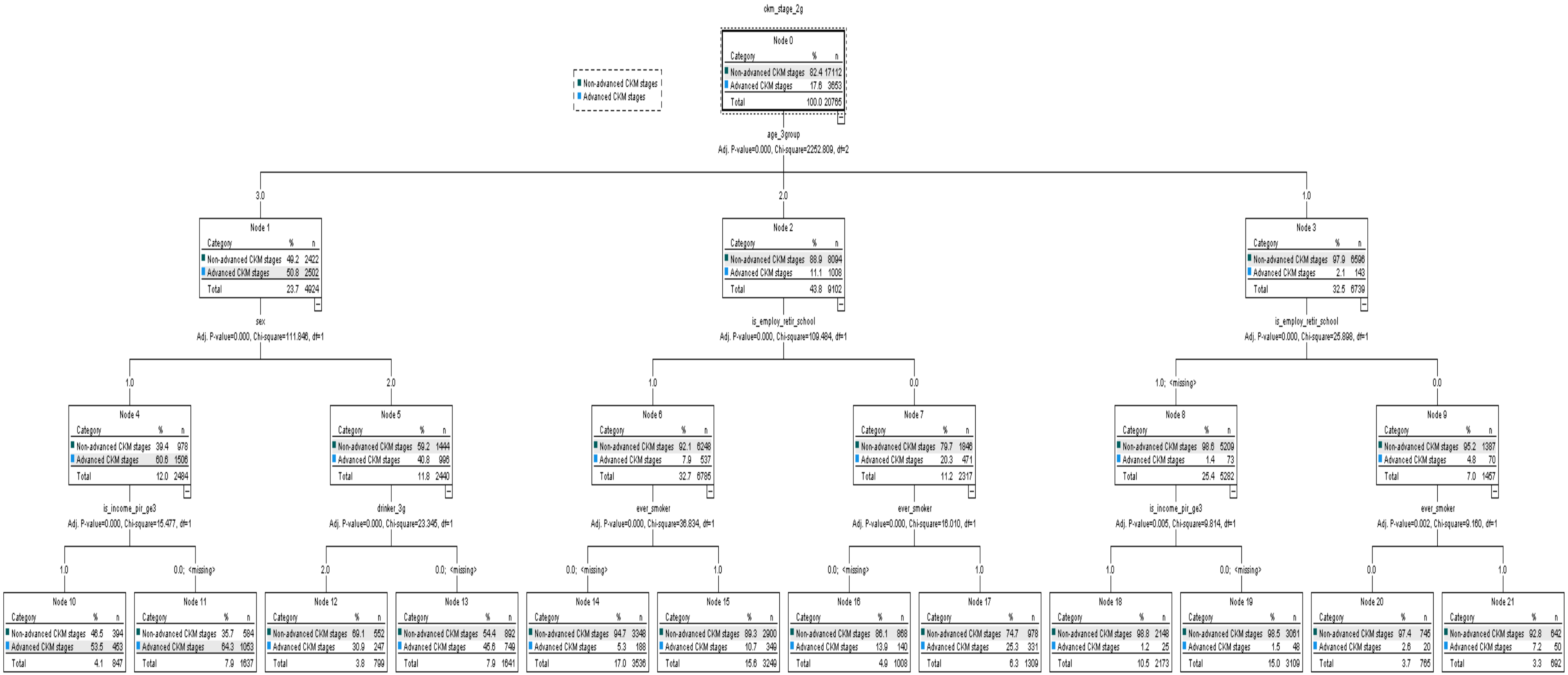

Abbreviation: CKM, cardiovascular-kidney-metabolic syndrome.

© 2024 Zhu R et al. *JAMA Network Open.*

eFigure 6. Decision Tree Created Using the Chi-Squared Automatic Interaction Detection Model for Advanced vs Nonadvanced Cardiovascular-Kidney-Metabolic Syndrome Stages, NHANES 2011-2018

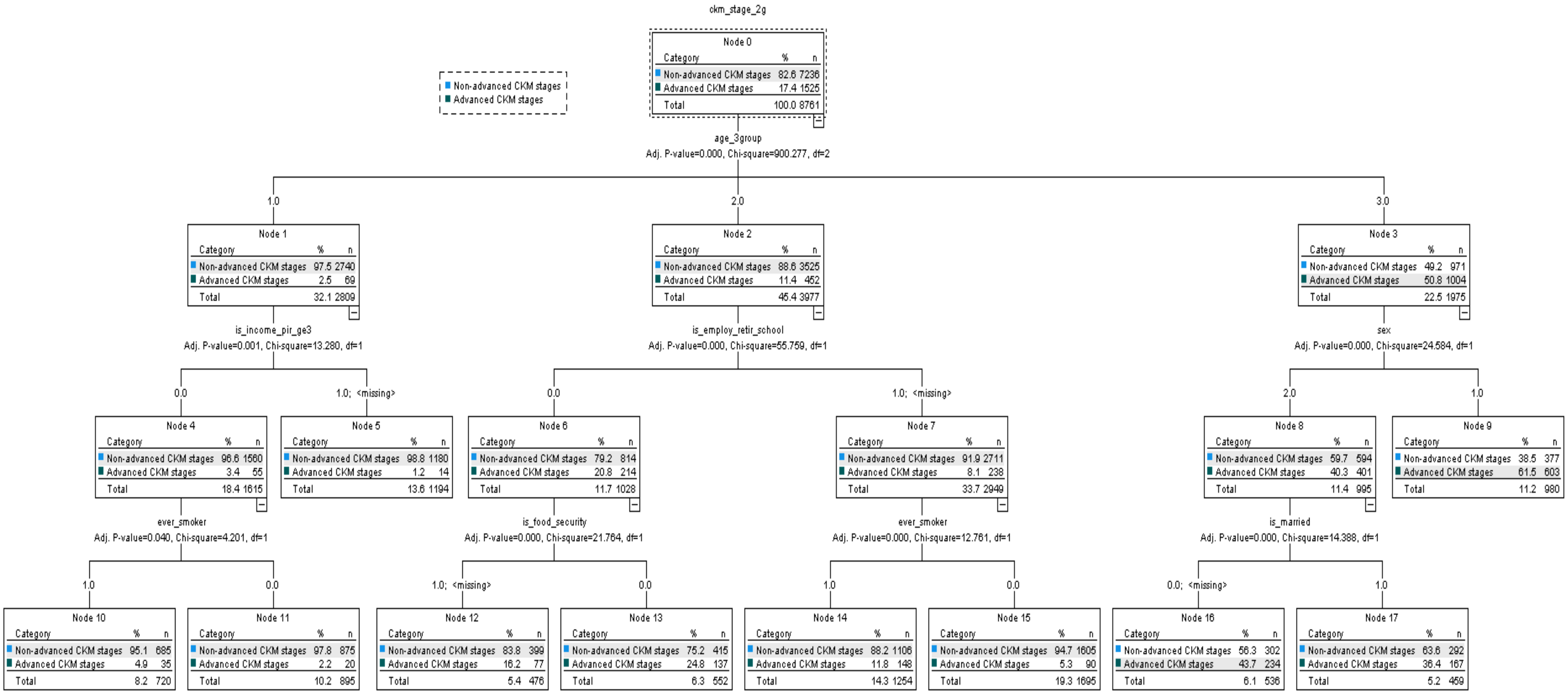

Abbreviation: CKM, cardiovascular-kidney-metabolic syndrome.

© 2024 Zhu R et al. *JAMA Network Open.*
