# Supplementary material for: Prevalence of Cardiovascular-Kidney-Metabolic Syndrome Stages by Social Determinants of Health
Source: JAMA Netw Open. 2024 Nov 18;7(11):e2445309. doi: 10.1001/jamanetworkopen.2024.45309 (PMC11574692; doi:10.1001/jamanetworkopen.2024.45309)
Supplement: Supplement 2. — Data Sharing Statement [file jamanetwopen-e2445309-s002.pdf]

Data Sharing Statement

Zhu. Prevalence of Cardiovascular-Kidney-Metabolic Syndrome Stages by Social Determinants of Health. *JAMA Netw Open*. Published November 18, 2024. doi:10.1001/jamanetworkopen.2024.45309

Data
Data available: Yes
Data types: Deidentified participant data
How to access data: Yes.All relevant data in this study are publicly available in National Health and Nutrition Examination Survey Homepage (https://www.cdc.gov/nchs/nhanes/index.htm). All- cause mortality was ascertained via linkage with the National Death Index (https://www.cdc.gov/ nchs/ndi/index.htm).
When available: With publication

Supporting Documents
Document types: Statistical/analytic code
How to access documents: Yes. The corresponding author Dr Jie Guo (email address: jie.guo@ki.se) should be contacted for any requests (e.g., data used for all analyses; analytic code; any other materials used in the current study).
When available: With publication

Additional Information
Who can access the data: Dr Jie Guo (email address: jie.guo@ki.se)
Types of analyses: Data used for all analyses; analytic code; any other materials used in the current study
Mechanisms of data availability: For any requests
